# Acceptability and willingness among men who have sex with men (MSM) to use a tablet-based HIV risk assessment in a clinical setting

**DOI:** 10.1186/2193-1801-3-708

**Published:** 2014-12-02

**Authors:** Jeb Jones, Rob Stephenson, Dawn K Smith, Lauren Toledo, Allison La Pointe, Jennifer Taussig, Patrick S Sullivan

**Affiliations:** Department of Epidemiology, Emory University, Atlanta, GA USA; Department of Global Health, Emory University, Atlanta, GA USA; Division of HIV/AIDS Prevention, Centers for Disease Control and Prevention, Atlanta, GA USA; ICF International, Atlanta, GA USA; Minnesota Department of Health, Saint Paul, MN USA

**Keywords:** HIV risk, Pre-exposure prophylaxis, HIV risk assessment, Men who have sex with men

## Abstract

We developed an iPad-based application to administer an HIV risk assessment tool in a clinical setting. We conducted focus group discussions (FGDs) with gay, bisexual and other men who have sex with men (MSM) to assess their opinions about using such a device to share risk behavior information in a clinical setting. Participants were asked about their current assessment of their risk or any risk reduction strategies that they discussed with their healthcare providers. Participants were then asked to provide feedback about the iPad-based risk assessment, their opinions about using it in a clinic setting, and suggestions on how the assessment could be improved. FGD participants were generally receptive to the idea of using an iPad-based risk assessment during healthcare visits. Based on the results of the FGDs, an iPad-based risk assessment is a promising method for identifying those patients at highest risk for HIV transmission.

## Background

Gay, bisexual, and other men who have sex with men (MSM) in the United States are disproportionately impacted by HIV. The proportion of HIV cases in the U.S. attributed to male-male sexual contact has increased steadily over the previous years, from 64% in 2006 to 73% in 2011 (Prejean et al. 2011). This growth has been most pronounced among young MSM, particularly young black MSM. From 2006–2009, new HIV infections increased 34% in young MSM overall and 48% among young black MSM (Prejean et al. 2011). Thus far, behavioral interventions alone have not been sufficient to end the epidemic. Combination interventions composed of behavioral and biomedical interventions might be key to reducing incidence (Sullivan et al. 2012).

Pre-exposure prophylaxis (PrEP) is a biomedical intervention that involves HIV-uninfected individuals taking specific antiretroviral drugs daily to reduce the risk of infection if exposure to HIV occurs. PrEP has been shown to reduce incidence of HIV in MSM and transgendered women by an average of 44%, including those with all levels of medication adherence (Grant et al. 2010). Among men with drug detected in their blood indicating recent adherence, PrEP reduced the rate of HIV acquisition by 92%. PrEP is recommended as one prevention option for MSM with “substantial risk” for HIV acquisition (U.S. Public Health Service 2014); thus, assessment of sexual behaviors is required to determine which MSM might be good candidates to receive PrEP. Although PrEP is a promising new biomedical prevention intervention, there are barriers to its implementation. PrEP is expensive to implement (Juusola et al. 2012); requires frequent clinic visits, often among patients who do not regularly engage with the healthcare system (Underhill et al. 2010); and identification of potential candidates for PrEP requires open and honest discussions between patients and healthcare providers about a patient’s sexual risk. The latter barrier is the focus of the current study.

Previous studies have shown that many MSM are reluctant to discuss their sexual orientation or sexual behaviors with their healthcare providers, and many providers believe they do not have the time to ask about, or do not feel comfortable discussing, same-sex sexual behavior with their patients (Bernstein et al. 2008; Meckler et al. 2006; Mimiaga et al. 2007; Petroll and Mosack 2011). In a sample of MSM in New York City, 39% reported never disclosing their sexual orientation to a health care provider, with nondisclosure highest among black and Hispanic MSM (Bernstein et al. 2008). In a sample of lesbian, gay, and bisexual youth in Los Angeles, only 35% had disclosed their sexual orientation to a physician (Meckler et al. 2006).

A patient’s sexual risk profile is essential to a clinician’s decision about whether or not to offer the patient PrEP or other intensive HIV prevention interventions. Inaccurate reporting of sexual risk due to clinician and/or patient discomfort contributes to missed opportunities for intervention. Potential candidates for PrEP are unlikely to be identified during a typical clinic visit in the absence of frank discussions about sexual risk-taking.

Cultural competency among clinicians in providing care to MSM is an important aspect of ensuring that the necessary discussions about sexual risk take place during a clinic visit. A recent survey of primary care physicians found that only half assessed a patient’s sexual history at least annually; far fewer asked patients about their sexual behavior at every visit (Wimberly et al. 2006). In another study of healthcare providers, only 17% asked about sexual preference when conducting a sexual history (Bull et al. 1999). However, physician experience with patients known to be MSM increases the frequency of sexual history-taking among medical students (Sanchez et al. 2006). Although the importance of cultural awareness and the identification of the unique healthcare needs of MSM patients have been recognized, efforts to increase the frequency of taking sexual history and evaluating risk for HIV during routine clinic visits are necessary (Epstein et al. 1998).

To help streamline the discussion of sexual behavior in the healthcare setting, to remove some of the social discomfort associated with such discussions, and to demonstrate a model to screen MSM for possible PrEP eligibility, we developed an iPad-based (i.e., an “app”) risk assessment to be used in healthcare settings to help identify potential PrEP candidates. The app contains questions about a patient’s behavioral sexual risk factors for HIV seroconversion, and provides a risk score that can be interpreted by a healthcare provider and used as the basis for further discussions about HIV risk and PrEP.

This exploratory study using focus group discussions (FGDs) with MSM examines the usability and acceptability of a tablet-based assessment in the healthcare setting. This information will help inform PrEP implementation by assessing acceptable methods for obtaining sexual risk information and attitudes about the use of technology in healthcare settings.

## Methods

### Participants

Three FGDs were conducted; two in Atlanta, GA in January 2010 (N = 5 and N = 9) and one in Minneapolis, MN in May 2010 (N = 10). Atlanta and Minneapolis were selected for their divergent geographic locations and HIV epidemics in order to obtain a variety of opinions and healthcare experiences. Eligible FGD participants were male at birth, ≥ 18 years of age, self-reported testing HIV negative, having had sex with a man in the past 12 months, being a resident in the Atlanta or Minneapolis metropolitan areas, and having had at least one visit to a healthcare provider in the past 12 months. Participants completed the FGDs anonymously, thus limited demographic and behavioral information (e.g., age, gender of sex partners in previous 12 months) was collected about each participant.

Participants in Atlanta had previously enrolled in a research study at Emory University (Sullivan et al. 2014) using venue-based time-space sampling (Muhib et al. 2001). Participants who indicated they were willing to be contacted for participation in future studies were contacted by email to complete an eligibility survey. Those meeting the eligibility criteria were invited to participate in one of the focus groups. Participants in Minneapolis were recruited via social media posts, internet advertisements, and a snowball method in which participants referred other potential participants to the study (Heckathorn 1997). Informed consent was obtained from all participants prior to participation in the FGD. This research was approved by the Emory University Institutional Review Board (IRB00046867).

### Focus group discussions

Focus group discussions followed a semi-structured interview guide and lasted approximately 45–60 minutes. FGDs were facilitated by one of the authors (JJ, RB, or PSS). The facilitator followed a discussion guide that prompted questions about interactions with healthcare providers, type of insurance coverage, disclosure of sexual behavior to healthcare providers, and opinions about the tablet-based risk assessment.

At the start of each discussion, participants described their interactions with the healthcare system, including their insurance status; how frequently they discussed their sexual orientation and/or sexual behaviors with their healthcare providers; and their level of comfort in these discussions with healthcare providers. Next, the iPad-based risk assessment tool was demonstrated for the group, and each individual was given a chance to complete the assessment using fictitious data of their choosing. The risk assessment consisted of nine questions regarding recent sexual behavior and three geographical questions (see the ‘Risk assessment items’ subsection). These questions were designed to obtain the zip codes of the participant, his most recent sex partner, and the place where they most recently had sex. HIV risk depends on an individual’s risk behaviors as well as the prevalence of HIV in their potential pool of sex partners. Thus, zip code data collection in an app would enable incorporation of local prevalence data with behavioral data to calculate a modified risk score. A discussion of participants’ opinions about the usability of the tablet-based assessment tool as well as their perceptions about completing a similar assessment in their healthcare providers’ office followed.

All FGDs were recorded using digital audio recorders. After each FGD, files were transferred to a computer and transcribed by one of the researchers (JJ) who was in attendance at each FGD.

### Risk assessment items

The iPad was programmed to administer a 12-item risk assessment with skip patterns. The items in the risk assessment were as follows:

1. Have you had sex in the past six months? [If yes, continue; if no, stop.]
2. Did you have sex with men, with women, or with both? [If men or both, continue; if women, stop.]
3. How old are you?
4. How many men have you had sex with in the past three months?
5. How many of your male sex partners were HIV positive?
6. In the past three months, how many times did you have receptive anal sex (you were the bottom) with an HIV-positive man?
7. In the past three months, how many times did you have receptive anal sex (you were the bottom) with a man whose HIV status you didn’t know?
8. In the past three months, have you used poppers, amyl nitrate, or ampules?
9. In the past three months, have you used methamphetamines such as crystal or speed?
10. What is your zip code?
11. What is your most recent sex partner’s zip code?
12. What is the zip code of the location where you most recently had sex?

### Coding and analysis

All FGD transcriptions were uploaded into NVivo, a qualitative data management and analysis software (QSR International Pty Ltd. Version 8 2008). A general inductive methodology was utilized to identify frequent, salient, and significant themes in the data (Thomas 2006). First, transcripts were structurally coded according to questions in the discussion guide; transcripts were then reviewed by structural code to identify concepts across focus groups using an iterative constant comparison approach (Guest and McLellan 2003; MacQueen et al. 1998). One researcher (LT) was primarily responsible for developing the codebook and coding the transcripts. Once coding was complete, codes were reviewed by interview question in order to identify prevalent themes both across and within focus groups for each section of the interview guide.

## Results

### Participants

Participant ages ranged from 20 to 46 years (median = 30 years). Overall, 14 white MSM and 10 black MSM participated in focus groups. One black participant reported having sex with men and women in the previous 12 months; the remainder reported sex with men only. The number of healthcare provider visits reported in the previous 12 months ranged from 1 to 8, with a median of 2 visits.

### Previous interactions with providers about HIV

Most participants had discussed sexual risk behaviors and HIV testing at some point with a healthcare provider. Both patient-initiated and provider-initiated discussions of HIV risk were reported. Patients who brought up HIV during a healthcare visit themselves typically did so by requesting to be tested for HIV. Typically, if a discussion about HIV risk was initiated by a provider it was conducted via structured questionnaires or conversations. Conversations ranged from recommendations to use condoms to conversations about specific sexual practices and the emotional causes of risk behavior. These discussions were largely reported to occur only during the first office visit or on specific visits, such as one following a potential sexual exposure to HIV, but not on a routine basis. As one participant explained,

Whose doctor really does that? I mean even with a good relationship or rapport built with your doctor. Who’s really sitting around talking about sex with their doctor unless it’s a gynecologist or a urologist, possibly. But, general practitioner? I mean unless [your appointment is] really about sex. Who’s really talking to their doctor about sex? Doctors are weirded out about the stuff, too.

In addition, many participants reported they had never disclosed the gender of their sexual partners to their healthcare provider(s).

Although most participants stated they were comfortable discussing sexual risk behaviors with their providers, this was not the case for all. One participant in particular who was enrolled in a large health maintenance organization (HMO) noted that he did not feel comfortable filling out the clinic risk questionnaire provided due to the size of the practice. This participant had the perception that too many different people had access to his medical records to want to answer questions honestly. Another participant noted that he would feel singled out if his healthcare provider discussed HIV risk at every visit:

If it’s not [a new behavior]…and [my doctor] doesn’t get any indication that I’m doing any risk behavior then I don’t want to hear that [risk reduction counseling]. [If] every time I come there they’re all over me, ‘Ahhhh, you’re doing that gay thing aren’t you? Having gay sex or something. That’s what it would equal; it would equal that in my head. So, unless I was doing something risky that they were aware of I wouldn’t want to hear that. Especially not every time I came.

The degree of comfort discussing sexual risk behaviors typically depended on the degree of comfort that a participant had with their provider rather than the provider’s job title (e.g., nurse, physician, etc.). Participants reported that they would be no more or less likely to disclose sexual risk behaviors to a nurse during an initial exam than to a physician.

### iPad-based risk assessment opinions

Opinions of the iPad-based risk assessment were generally very positive. Overall, the risk assessment was regarded as “quick and easy to do” and participants indicated a high level of willingness to complete a similar screening during a healthcare provider visit. Although opinions varied on a maximum acceptable length for the risk assessment, all agreed that the prototype tested during the FGD was an acceptable length (approximately three to five minutes in length).

Participants indicated that the app might have benefits beyond communicating their sexual risk to providers:

I think that it’s good just to start yourself thinking about what you have been doing before you go into your doctor in case you remember a question you had or it jogs your memory about something you might want to ask.

The men also suggested that they would like for the risk assessment to include other talking points to bring up with their provider because they did not always know which questions to ask. For example, participants thought it would be beneficial if the app provided information about screening tests they may need, or HIV/STD prevention programs available in the area. Many participants were also open to the idea of entering their e-mail address so that they could receive personalized sexual risk management information based on their answers to the assessment. Some men noted that receiving the assessment by e-mail would allow them to privately review the information after their visit.

Participants suggested adding questions to the tablet-based tool regarding the following topics: alcohol and drug use; relationship status with sex partner; demographic characteristics of partner; information on where the patient meets his sex partners; type of sex engaged in with different partners (e.g., oral, anal receptive, anal insertive); and use of condoms and/or lubricant.

### Preferences regarding administration

Most participants either preferred the iPad-based method to a paper and pencil questionnaire, or expressed no preference between the two methods. The iPad-based risk assessment was perceived to be more private and secure, environmentally friendly, and more accessible for younger patients. In contrast, an older participant worried that patients his age and older might be distrustful of the technology and hesitant to use it, preferring instead to talk face-to-face with their provider.

Most participants reported that they would prefer to self-administer the risk assessment in the waiting room rather than have a provider ask the questions. These participants felt this would make it easier to honestly disclose their sexual behavior. One participant said,

I would rather [fill it out myself] than talk to [the doctor] if I didn’t know him. It would cut down my nervousness on having to see my doctor’s face if the reaction was not going to be good.

Another participant noted:

I would rather fill it out. I remember when I started going to the testing clinics, you don’t really know these people, so I know I used to tell a few stories.

The idea of the provider administering the risk assessment seemed puzzling to many participants. Participants indicated that they would be confused about why the provider needed an iPad to ask that series of questions. In addition, the amount of time available with a physician was perceived to be limited, so as one participant said:

I [would] want to fill it out before [I see the doctor], to take up time in the waiting room… If I’m with the doctor I’d rather just tell him rather than sitting here with an iPad while I’m trying to talk to him.

Filling out the risk assessment themselves was perceived to speed up the visit and maximize already limited time available for the visit. However, some participants were worried that the provider would not look at the results until they were in the examination room, even if the patient filled it out in advance.

### Benefits and barriers of iPad-based risk assessment

All participants reported that an iPad-based risk assessment would result in responses to sensitive questions that were just as honest as or more honest than the same questions being asked face-to-face. Additionally, there was a strong perception that this tool would facilitate difficult conversations with the provider about HIV risk and sexual behavior. Although participants felt the iPad-based tool would not negatively impact patients’ honesty, the men did identify some barriers to using the tool accurately and effectively. Participants viewed the geographical questions as excessively intrusive and difficult to answer by all participants. Most suggested they would have no way of knowing anyone’s zip code other than their own.

Other barriers emerged after the moderator explained PrEP and the purpose of the risk assessment tool. Many participants were concerned that there would be “PrEP-seekers” who would lie about their behavior in order to try to control the recommendations made by the risk assessment. One participant echoed the sentiment of many saying,

If there was anything that let you know it was being used that way it could change how people answer. Because someone could be like, ‘oh, well, I want to try to get those meds reduced and get them at a better price. Maybe if I put answers to really make myself look risky then I might qualify for some.’

Some participants were put off at the idea of being handed a risk assessment that they felt singled them out as an MSM at their healthcare provider’s office. Particularly among black participants, there was a perception that if they, but not all participants, were handed a risk assessment that asked about engaging in sex with other men then they would feel differentially targeted compared to the patients perceived to be heterosexual. Other participants, however, disagreed and felt that their provider would be looking out for their best interests.

## Discussion

The idea of an iPad-based HIV-risk assessment to be used in healthcare provider offices was well received by the MSM in three focus groups. Additionally, the prototype tested was regarded as user-friendly and participants indicated that it would be a welcome addition if implemented in their own healthcare provider’s office. Although most participants had discussed their HIV risk behaviors in some form with their provider, virtually all indicated that this was not a regular topic of discussion. In fact, many participants had never disclosed the gender of their sexual partners to their healthcare providers.

Although most participants were comfortable discussing sexual risk behaviors with their providers, this was not the case for all. This suggests that patients should be provided with options for completing the risk assessment based on what makes them most comfortable. Further, social desirability bias might affect patients’ responding. Sexual history reporting has been shown to vary based on the method of administration in research settings (e.g., Metzger et al. 2000) and in clinical settings (Ghanem et al. 2005; Des Jarlais et al. 1999; Locke et al. 1992, 1994; Robinson and West 1992; Kurth et al. 2004). For example, in the study by Ghanem et al. ( 2005), sexual risk behaviors were underreported by patients in a STD clinic when assessed via face-to-face interview compared to audio computer assisted self-interview (ACASI). Kurth et al. ( 2004) found lower reporting of same-sex sexual behavior in male participants when sexual history was taken by a clinician compared to ACASI. The effectiveness of a screening tool for HIV risk is dependent on full disclosure of sexual risk by patients. Self-administration of the risk assessment might result in more valid responses compared to a provider-administered assessment.

The FGD participants had many suggestions of additional questions to add to the risk assessment. Even if these questions are not factored into the risk algorithm, a patient’s answers could provide valuable information to the healthcare provider in terms of targets for HIV risk counseling. Any additional questions will increase the time needed to complete the risk assessment and this will need to be considered if a risk assessment is implemented in clinical practice.

Age differences were also a frequent topic of discussion. Both younger and older participants thought that the iPad-based risk assessment would be more acceptable among younger patients than older patients. More specifically, there was a perception that older patients would be distrustful of the technology whereas younger patients would likely be more honest when answering questions on an iPad compared to face-to-face with a healthcare provider. A recent study, however, indicates that older adults have generally positive attitudes towards using technology in a healthcare setting (Mitzner et al. 2010).

Assessment of geographic information via zip codes was viewed as excessively intrusive, and participants indicated that they would be unlikely to know any zip codes other than their own. If geographic information is incorporated, it may be best to use a less specific geographic indicator than zip code. Geographic information regarding place of residence of the participant, the participant’s sex partner(s), and place of last sex might also be obtained using a map tool that is integrated into the risk assessment. A recent study found that most men were able to pinpoint their own address and their healthcare provider’s office to within one mile on a map (Dasgupta et al. 2014).

This study has limitations. We conducted a limited number of focus groups with convenience samples of MSM in two cities and did not achieve saturation. We were unable to recruit a FGD of black MSM in Minneapolis, and we only had three FGDs overall. Although it would have been desirable to have more FGDs, the opinions and views expressed were consistent across the FGDs and geographic locations, indicating broad consensus regarding the acceptability and usability of a tablet-based risk assessment. The purpose of this study was to conduct a preliminary examination of attitudes regarding iPad-based risk assessments in a clinical setting, and the results of this study must be interpreted accordingly. FGD participants demonstrated a willingness to disclose same-sex sexual behavior as a condition of eligibility and many already discussed sexual risk behaviors with their healthcare providers. It is unclear how well the opinions of these men about the acceptability of the tablet-based screener will generalize to other populations. We recognize that it will also be important to collect, analyze, and report similar data on willingness from healthcare providers to use a tablet-based device in their practice. Future studies should also investigate the extent to which a tablet-based risk assessment identifies new high-risk patients and increases provider knowledge about their patients’ sexual risk behaviors as well as the degree to which social desirability might influence patients’ responses.

A tablet-based risk assessment will be most effective if providers are given appropriate training on cultural competency and the broad array of behavioral and biomedical prevention options available. Culturally competent care for MSM is often lacking in the United States (U.S. Department of Health and Human Services 2013); however, it is necessary in order to assure that physicians or other health care providers do not miss opportunities to recommend HIV prevention interventions such as PrEP to eligible patients. Healthcare providers often lack the skills or training to conduct sexual risk assessments (Krakower and Mayer 2012). Further, uptake of PrEP among providers is low. In one survey, only 4% of providers reported ever prescribing PrEP (White et al. 2012). An iPad-based risk assessment would offer the benefit of streamlining the discussion of sexual health and behavior while at the same time reducing some of the discomfort experienced by both patients and providers. Additionally, the use of a computer program allows for the standardization of recommendations based upon a patient’s particular risk profile, potentially filling a gap in provider knowledge. This type of application, in which an established decision rule is applied routinely and broadly, has been identified as an ideal application of technology in HIV prevention for MSM (Sullivan et al. 2013). A number of HIV risk assessments have been published (e.g., Kahle et al. 2013; Smith et al. 2012) for different populations, and we expect that the general feedback on acceptability would be similar with these measures.

The fight against HIV must be a multifaceted one, and the use of an iPad-based risk assessment to identify risk and engage healthcare providers in conversation about HIV risks with their patients may be an important component of a clinic-based comprehensive HIV risk reduction program. The ability to help providers identify those patients most in need of intensive interventions such as PrEP, and to remind the provider and the patient to discuss sexual health and behavior are two crucial benefits provided by this technology.

## Abbreviations

FGD: Focus group discussion
MSM: Men who have sex with men
PrEP: Pre-exposure prophylaxis.
